# Inertial Measurement Units and Application for Remote Health Care in Hip and Knee Osteoarthritis: Narrative Review

**DOI:** 10.2196/33521

**Published:** 2022-06-02

**Authors:** Michael J. Rose, Kerry E Costello, Samantha Eigenbrot, Kaveh Torabian, Deepak Kumar

**Affiliations:** 1 Department of Physical Therapy & Athletic Training Boston University College of Health & Rehabilitation Sciences: Sargent College Boston, MA United States; 2 Division of Rheumatology, Department of Medicine Boston University School of Medicine Boston, MA United States

**Keywords:** wearable technology, accelerometer, gyroscope, magnetometer, remote monitoring, biofeedback

## Abstract

**Background:**

Measuring and modifying movement-related joint loading is integral to the management of lower extremity osteoarthritis (OA). Although traditional approaches rely on measurements made within the laboratory or clinical environments, inertial sensors provide an opportunity to quantify these outcomes in patients’ natural environments, providing greater ecological validity and opportunities to develop large data sets of movement data for the development of OA interventions.

**Objective:**

This narrative review aimed to discuss and summarize recent developments in the use of inertial sensors for assessing movement during daily activities in individuals with hip and knee OA and to identify how this may translate to improved remote health care for this population.

**Methods:**

A literature search was performed in November 2018 and repeated in July 2019 and March 2021 using the PubMed and Embase databases for publications on inertial sensors in hip and knee OA published in English within the previous 5 years. The search terms encompassed both OA and wearable sensors. Duplicate studies, systematic reviews, conference abstracts, and study protocols were also excluded. One reviewer screened the search result titles by removing irrelevant studies, and 2 reviewers screened study abstracts to identify studies using inertial sensors as the main sensing technology and a primary outcome related to movement quality. In addition, after the March 2021 search, 2 reviewers rescreened all previously included studies to confirm their relevance to this review.

**Results:**

From the search process, 43 studies were determined to be relevant and subsequently included in this review. Inertial sensors have been successfully implemented for assessing the presence and severity of OA (n=11), assessing disease progression risk and providing feedback for gait retraining (n=7), and remotely monitoring intervention outcomes and identifying potential responders and nonresponders to interventions (n=14). In addition, studies have validated the use of inertial sensors for these applications (n=8) and analyzed the optimal sensor placement combinations and data input analysis for measuring different metrics of interest (n=3). These studies show promise for remote health care monitoring and intervention delivery in hip and knee OA, but many studies have focused on walking rather than a range of activities of daily living and have been performed in small samples (<100 participants) and in a laboratory rather than in a real-world environment.

**Conclusions:**

Inertial sensors show promise for remote monitoring, risk assessment, and intervention delivery in individuals with hip and knee OA. Future opportunities remain to validate these sensors in real-world settings across a range of activities of daily living and to optimize sensor placement and data analysis approaches.

## Introduction

### Background

Delivery of care and assessment of outcomes in patients’ natural environments have made large strides in recent years. The COVID-19 pandemic has further created a need for and accelerated the adoption of remote approaches to health care. Wearable sensors, which are used to describe small, lightweight measurement devices that can be worn on the body [1], have become integral to models of remote care and assessment. These devices can be worn directly on the body or within an accessory (eg, a watch) without altering the user’s natural behavior.

Osteoarthritis (OA) is a mechanically driven disorder and a leading cause of disability in middle-aged and older age adults [2]. The burden of OA is primarily due to the greater prevalence of knee and hip OA [3], including those who undergo joint replacement surgery for hip or knee OA [4]. In people with hip or knee OA, abnormal joint loading during daily activities has been associated with pathogenesis [5], driving interest in assessing the relationships between repetitive loading during everyday movements and disease outcomes and interventions to alter these loads [6]. Although there is a large body of literature on understanding movement patterns during daily activities in people with knee or hip OA, a majority of the prior work has used laboratory or clinical assessments, which have limited ecological validity [7]. Furthermore, the gold standard for measuring human movement, optical motion capture, requires expensive equipment, skilled technicians, and a large calibrated measurement space, limiting its deployment on a large scale. In contrast, wearable technology can provide large volumes of data from real-world settings with relative ease. These data could improve health care quality by allowing remote monitoring to inform treatment planning [8], for remote care delivery to address provider and patient time constraints [9], and for promoting active patient engagement through actionable insights [9]. Thus, wearable sensors offer tremendous opportunities to advance research and care for people with hip or knee OA, including those who undergo joint replacement surgery, most frequently total hip arthroplasty (THA) or total knee arthroplasty (TKA) of the arthritic joint.

The most common wearable movement sensors that have been used for OA applications are accelerometers, gyroscopes, and magnetometers. Accelerometers measure the applied acceleration (ie, rate of change of linear velocity) along a sensitive axis [10]. Gyroscopes measure angular velocity (ie, the rate of change of angular motion) within a rotating reference frame [11]. Magnetometers capture data that can provide heading information, including body orientation, by sensing Earth’s gravitational field [12]. All 3 of these sensors have limitations: accelerometers suffer from signal drift [13], poor reliability in measuring nondynamic events [14], and the impact of gravity on acceleration signals [12]; gyroscopes experience problems with drift, particularly during turning movements [11]; and magnetometers can be affected by other magnetic fields (eg, nearby ferromagnetic objects) [15]. Consequently, these technologies are often used in combination, especially as inertial measurement units (IMUs; also known as *inertial sensors*), consisting of an accelerometer, gyroscope, and sometimes a magnetometer. Inertial sensors are relatively inexpensive, small, lightweight, and unobtrusive, allowing for implementation in large cohorts; these sensors can be used alongside other technologies or types of sensors to provide feedback to users (eg, mobile apps).

### Objectives

The aim of this review was to analyze the current uses and limitations of using inertial sensors for assessing movements during daily activities in individuals with hip or knee OA, including those who undergo joint replacement surgery, and to identify how this may translate to improved remote health care in this population. We conclude with a discussion highlighting the potential future applications and remaining areas where further development is required. This review may be used to inform current practices and further research on these promising technologies.

## Methods

### Search Strategy

For this narrative review, we performed an initial literature search in the PubMed and Embase databases in November 2018 and repeated the search in July 2019 and March 2021. The keywords used for the search were (“IMU” OR “inertial sensor” OR accelerometer OR gyroscope OR magnetometer OR wearable* OR sensor) AND (osteoarthritis OR arthritis OR “TKR” OR “TKA” OR “knee replacement” OR “knee arthroplasty”).

### Data Extraction

We included studies that met the following criteria: (1) original studies published in the English language, (2) published within the previous 5 years, (3) used inertial sensors for the study of human movement, and (4) included data from people with OA or those with knee replacement. We excluded studies that used inertial sensors to study other related constructs (eg, sleep quality and physical activity) but did not directly study movement patterns. We excluded studies that focused on individuals with knee injuries without a diagnosis of knee OA (eg, anterior cruciate ligament tear and meniscus injury). Duplicate studies, systematic reviews, conference abstracts, and study protocols were also excluded. One researcher (either SE or MJR) screened the search result titles, removing studies that were not relevant to this review. For the remaining studies, 2 researchers (either SE and DK or MJR and KEC) read each study abstract to determine whether the study should be included. The final decision on inclusion was made in consensus by MJR, KEC, and DK. A total of 2 authors (SE and MJR) reviewed the included studies and annotated key information, including study objective, study population, details of the inertial sensor, specifics of the application for which the sensors were used, and the findings. After reviewing this information, we categorized the included studies based on the study objective to organize this review for the reader. Specifically, we categorized the studies into those related to the validity and repeatability of inertial sensor measurements (n=8), assessment of OA presence and severity (n=11), assessment of movement patterns associated with OA progression and gait retraining (n=7), assessment of OA intervention outcomes (n=14), and sensor placement and data analysis (n=3). For each of these sections, we synthesized the findings from the included studies with a focus on applications, limitations, challenges, and possible future directions. Tables are presented for each section, summarizing the key information from the included studies. Detailed descriptions of the study and sensor applications can be found in tables in Multimedia Appendix 1 [16-56].

## Results

**Figure 1:**
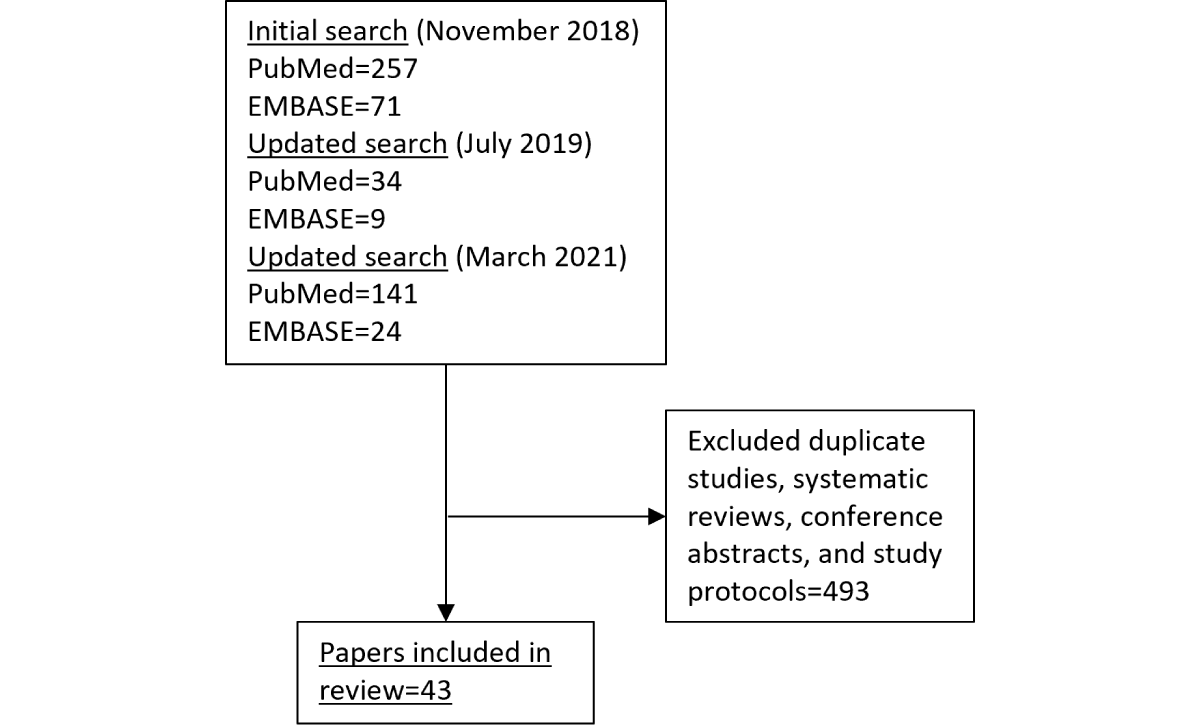
Literature search process.

Our literature search identified a total of 536 papers, of which 43 were determined relevant and included in this review (Figure 1).

### Validity and Repeatability of Inertial Sensor Measurement of Movement

As the use of wearable technology for movement quality assessment has increased, there is a need to assess the repeatability and validity of these technologies (Table 1; Table S1 in Multimedia Appendix 1). In people with hip OA, waveforms recorded from a single pelvic IMU were reported to have a shape and magnitude similar to those recorded by optical motion capture [57]. Using a robotic arm and anthropomorphic leg phantom to simulate knee flexion at 3 different speeds, Fennema et al [16] identified acceptable test-retest repeatability of IMU-based joint angle measurements (<+5° or −5°) across different knee flexion speeds or with repositioning of the IMUs. In healthy young adults, the foot progression angle (FPA), that is, the angle of the foot relative to the direction of travel, has also been measured with good to excellent validity (intraclass correlation coefficient=0.89-0.91) and reliability (intraclass correlation coefficient=0.95) [17] and with errors <2° compared with optical motion capture [58] using a shoe-embedded IMU. Many IMU systems have been successfully validated against optical motion capture, including a 17-IMU system used to estimate knee adduction moment (KAM) and tibiofemoral joint contact forces [18]; a 4-IMU system used to measure spatiotemporal gait variables and knee range of motion (ROM) [19]; and a 7-IMU system used to measure ankle, knee, and hip joint angles in populations with hip [20] and knee OA [21]. In addition, Bravi et al [22] found a single, lower trunk IMU valid for measuring spatiotemporal gait parameters in both healthy participants and patients with recent TKA or THA walking with crutches; however, the device struggled with gait cycle phase recognition in the patient group. Youn et al [59] found that variables related to initial loading behavior (ie, knee flexion moment, KAM, anterior ground reaction force, and vertical ground reaction force) could be predicted (*R*^2^≥0.60) from 10 temporal and kinetic parameters extracted from 2 ankle-worn accelerometers in patients post TKA. These studies suggest that wearable sensors can be used to estimate joint kinetics. IMU-based systems have also been found to provide valid metrics compared with optical motion capture during more demanding tasks (ie, stair ascent, stair descent, and sit-to-stand) in healthy older adults [18] and during level walking in individuals post THA [60]. Furthermore, low coefficient of variance values (<10%) was reported when IMUs were placed by different operators or when sensors were displaced along the anteroposterior and mediolateral axes by +20 to −20 mm [23]. As hardware enhancements continue and with the availability of larger data sets, it is anticipated that the performance of these devices will continue to improve, particularly with the use of advanced machine learning approaches for data analysis.

**Table 1:** Inertial sensors validity and reliability measuring movement.

Study	Population	Sensor	Findings
Bravi et al, 2020 [22]	Healthy (n=10), THA^a^ (n=10), and TKA^b^ (n=10)	Single IMU^c^ (G-WALK, BTS Bioengineering) on trunk	Single IMU reliable for measuring spatiotemporal gait in individuals using crutches
Charlton et al, 2019 [17]	Healthy (n=20)	Single IMU^c^ (MPU-9150, InvenSense) embedded in shoe sole under heel	Good to excellent reliability measuring foot progression angle in overground walking
Fennema et al, 2019 [16]	Anthropomorphic phantom leg	2 IMU^c^ (MetaMotionR, mbientlab) on thigh and shank	Acceptable repeatability in range of motion measurements from 2 different IMU placements
Hafer et al, 2020 [19]	Healthy (n=20) and knee OA^d^ (n=9)	4 IMU^c^ (OPAL, APDM) on foot, shank, thigh, and lower back	Minimal IMU setup and reproducible methods can accurately capture gait metrics
Ismailidis et al, 2020 [20]	Healthy (n=45) and hip OA (n=22)	7 IMU^c^ (RehaGait, Hasomed) on pelvis, feet, shanks, and thighs	Validated commercial IMU system against literature on marker-based data differences between hip OA and healthy individuals
Ismailidis et al, 2021 [21]	Healthy (n=46) and knee OA (n=22)	7 IMU^c^ (RehaGait) on pelvis, feet, shanks, and thighs	Sensors able to discriminate between knee OA and healthy individuals and between affected and unaffected sides in unilateral knee OA
Konrath et al, 2019 [18]	Healthy (n=8)	17 IMU^c^ (Xsens Awinda, Xsens Technologies BV) on entire body	Moderate to strong Pearson correlation coefficients found between knee adduction moment and tibiofemoral joint contact force calculations
Zügner et al, 2019 [60]	THA (n=49)	6 IMU^e^ (GaitSmart, Dynamic Metrics Ltd) on iliac crests, thighs, and shanks	Validated IMUs for measuring mean pelvic tilt and knee flexion angles

^a^THA: total hip arthroplasty.

^b^TKA: total knee arthroplasty.

^c^IMU: inertial measurement unit (with accelerometer, gyroscope, and magnetometer).

^d^OA: osteoarthritis.

^e^IMU with accelerometer and gyroscope.

### Assessment of OA Presence and Severity

One of the most common applications of inertial sensors identified in this review was to determine the presence or severity of hip or knee OA using IMU-derived movement parameters (Table 2; Table S2 in Multimedia Appendix 1). Across these studies, there was a wide variation in the methods used to extract various movement parameters. Simpler approaches rely on using raw sensor data and focus on walking gait. For instance, Tanimoto et al [24] compared the peak shank angular velocity during swing directly measured from a gyroscope between people with knee OA and controls. The authors determined gait cycles using an acceleration signal. Although they did not find any significant differences in the average and variability measures of peak shank angular velocity between groups, they observed that greater angular velocity and lower variability of peak angular velocity were related to lower pain and better participant-reported function. Another relatively simple approach included using the mean and root mean square of the acceleration and angular velocity signals from foot-worn IMUs without undertaking any gait cycle detection [23]. Using this approach, Barrois et al [23] identified 4 of 61 parameters to be discriminative between people with knee or hip OA with moderate impairments, those with severe impairments, and healthy controls. However, given the large number of comparisons with a relatively small sample and no adjustment of the *P* value, their findings may be susceptible to type 1 errors. Finally, Na et al [25] reported a greater magnitude of tibial acceleration and tibial jerk (ie, the time derivative of acceleration) during the midstance phase of walking in people with knee OA compared with controls and greater acceleration being related to greater self-reported knee instability. The findings from these studies suggest that information extracted from the raw acceleration or angular velocity signals, even from a single sensor, may be useful to discriminate between people with knee OA and controls and could be related to clinically meaningful participant-reported outcomes.

**Table 2:** Inertial sensors and assessment of osteoarthritis presence and severity.

Study	Population	Sensor	Findings
Barrois et al, 2016 [23]	Healthy (n=12) and knee or hip OA^a^ (n=48)	4 IMU^b^ (MTw, Xsens Technologies BV) on feet, lower back, and head	Found discrimination capacity between OA severity groups in parameters of mean and root mean square of horizontal acceleration in both feet
De Brabandere et al, 2020 [31]	Hip OA (n=20)	Single IMU^c^ (Samsung Galaxy J5 2017, Samsung) inside cell phone, attached to hip	Trained machine learning pipeline to estimate hip and knee joint loading; error too large for clinical use
Dindorf et al, 2020 [32]	Healthy (n=27) and THA^d^ (n=20)	7 IMU^c^ (Awinda, Xsens Technologies BV) on feet, shanks, thighs, and back	Automatically extracted features gave best machine learning accuracy in discriminating THA from healthy individuals
Ismailidis et al, 2020 [61]	Healthy (n=48) and hip OA (n=24)	7 IMU^b^ (RehaGait, Hasomed) on pelvis, feet, shanks, and thighs	Significant changes in hip and knee kinematics exist between hip OA and healthy individuals in speed matched conditions
Ismailidis et al, 2020 [26]	Healthy (n=28) and knee OA (n=23)	7 IMU^b^ (RehaGait) on pelvis, feet, shanks, and thighs	Significant differences in all spatiotemporal parameters between groups when walking at self-selected speed
Na and Buchanan, 2021 [25]	Healthy (n=13) and knee OA (n=26)	5 IMU^c^ (3D myoMOTION, Noraxon) on pelvis, thighs, and shanks	Linear acceleration (significant) and jerk (insignificant) negatively associated with self-reported instability
Odonkor et al, 2020 [27]	Healthy (n=10) and knee OA (n=10)	2 IMU^b^ (Shimmer3, Shimmer Sensing) on feet	Stance and double support ratio 2 most consistent discriminating features between OA and controls
Tadano et al, 2016 [28]	Healthy (n=8) and knee OA (n=10)	7 IMU^c^ (H-Gait system, Laboratory of Biomechanical Design, Hokkaido University) on pelvis, thighs, shanks, and feet	Angle between knee trajectories nearly twice as large in OA individuals compared with healthy controls
Tanimoto et al, 2017 [24]	Healthy (n=11) and knee OA (n=12)	Single IMU^c^ (MVP-RF8-GC-500, Microstone) on anterior shank	No differences between 2 groups for any parameters for peak shank angular velocity
Van der Straaten et al, 2020 [29]	Healthy (n=12) and knee OA (n=19)	15 IMU^b^ (MVN BIOMECH Awinda) on entire body	Individuals with knee OA walked with significantly less trunk rotation, less internal pelvic rotation during stance to swing, and reduced knee flexion among other discriminating differences
Van der Straaten et al, 2020 [30]	Healthy (n=12) and knee OA (n=19)	15 IMU^b^ (MVN BIOMECH Awinda) on entire body	Knee OA individuals had more lateral trunk lean toward contralateral leg and more hip flexion throughout performance of unipodal stance task

^a^OA: osteoarthritis.

^b^IMU: inertial measurement unit (with accelerometer, gyroscope, and magnetometer).

^c^IMU with accelerometer and gyroscope.

^d^THA: total hip arthroplasty.

Other studies have used more computationally complex approaches to extract spatiotemporal parameters and joint kinematics during walking using IMU data. Ismailidis et al [26,61] published 2 studies, one each in people with end-stage hip OA and those with end-stage knee OA, in which they compared spatiotemporal and sagittal plane kinematics from IMUs between OA and control populations. Using statistical parametric mapping, they observed differences in multiple parameters (eg, cadence, knee, and hip kinematics) between each OA population and controls. Differences in spatiotemporal parameters between people with knee OA and controls [27] and in joint kinematics among knees with varying OA severity [28] have also been reported by other studies. These approaches are closer to the information traditionally obtained using 3D motion capture systems and allow for comparisons with existing literature. However, most of these studies relied on commercial systems, which raises concerns about the accuracy and validity of the data because the algorithms tend to be proprietary.

In addition to walking, IMUs were used to compare movement patterns during other daily activities between individuals with OA and controls. In 2 studies from the same cohort of people with end-stage knee OA and controls, van der Straaten et al [29,30] compared movement patterns during various activities, including walking, lunge, stair climbing, squatting, sit-to-stand, and single-leg balance. They reported differences in multiple measures, including those representing motions of the trunk and pelvis, which had not been previously reported. These authors also used a commercial system but undertook a validation study against optical motion capture. They concluded that the given IMU system was not ready for the assessment of movement patterns in patients with knee OA, particularly for motions in the frontal plane.

The final 2 studies in this section used machine learning approaches during the postprocessing of IMU data [31,32]. Going beyond spatiotemporal parameters and joint kinematics, De Brabandere et al [31] estimated hip and knee contact forces during various daily activities from a single IMU within a smartphone using machine learning. They observed differences in the model performance across joints (hip vs knee) and activities. They concluded that their approach, which was easy to use and promising in terms of model performance, did not result in an estimate of contact force that was sufficiently accurate for clinical use. However, this study represents an important advancement in the estimation of joint contact forces from IMUs, and future work with multiple sensors and more advanced machine learning approaches may yield better results. Finally, Dindorf et al [32] used explainable artificial intelligence to classify people into those post total hip replacement and controls using data from 7 IMUs during walking. They used both raw data and joint kinematic data as inputs in different models and observed excellent model performance. They reported that sagittal movement of the hip, knee, and pelvis, along with transversal movement of the ankle, was especially important for classification [32]. The use of machine learning and deep learning approaches is only expected to increase, particularly as IMUs facilitate the collection of data in cohorts much larger than is possible with traditional motion capture. These approaches could eventually lead to digital biomarkers of OA from data collected using simple and inexpensive IMU sensors.

### Assessment of Movement Parameters Related to OA Progression and Gait Retraining

Although discriminating between people with and without OA is important, being able to identify individuals at risk of worsening disease early in the disease process would be even more valuable. To this end, another key application of inertial sensors was in using these relatively low-cost sensors to quantify important gait parameters that have previously been associated with knee OA progression (Table 3; Table S3 in Multimedia Appendix 1), such as varus thrust, KAM [62,63], and FPA [64]. Capturing these parameters would traditionally require expensive 3D motion capture technologies, but inertial sensors may allow these risk factors to be captured with relative ease and at low cost in large samples.

**Table 3:** Inertial sensors and assessment of movement patterns associated with osteoarthritis progression and gait retraining.

Study	Population	Sensor	Findings
Costello et al, 2020 [33]	Knee OA^a^ (n=26)	3 IMU^b^ (Trigno IM Sensors, Delsys Inc) on thigh, midshank, and distal shank	Single-leg sensor metrics were associated with surrogate measures of varus thrust, and midthigh adduction velocity was significantly associated with peak external knee adduction moment
Ishii et al, 2020 [34]	Knee OA (n=44)	2 IMU^c^ (WAA-010, ATR-Promotions) placed on tibia and foot	Positive correlation between lateral thrust and change in medial meniscus extrusion
Iwama et al, 2021 [35]	Knee OA (n=22)	6 IMU^c^ (TSND151, ATR-Promotions) on pelvis, sternum, shanks, and thighs	Moderate correlation found between acceleration peak in IMU frame and KAM, values from shank IMU had strongest correlation
Karatsidis et al, 2018 [38]	Healthy (n=11)	7 IMU^b^ (MTw, Xsens Technologies BV) on pelvis, thighs, shanks, and feet	High accuracy and repeatability of foot progression angle measures, and feedback effectiveness was similar between wearable and laboratory feedback setups
Wang et al, 2020 [36]	Healthy (n=12), knee OA (n=78)	2 IMU^c^ (DA14583, Dialog Semiconductor) on malleoli	Two machine learning algorithms were highly accurate (*R*^2^ approximately 0.95) in predicting KAM using IMU input
Wouda et al, 2021 [37]	Healthy (n=5)	2 IMU^b^ (MTw Awinda, Xsens Technologies BV) on feet	Good correlation coefficients to discriminate between different foot progression angle walking conditions
Xia et al, 2020 [39]	Healthy (n=10)	Single IMU^b^ (custom-made) embedded in shoe sole	Participants were able to respond to feedback during walking and adopt target foot progression angle conditions

^a^OA: osteoarthritis.

^b^IMU: inertial measurement unit (with accelerometer, gyroscope, and magnetometer).

^c^IMU with accelerometer and gyroscope.

Different sensor configurations during walking have been used to quantify varus thrust in gait; and one study using sensors on the thigh, midshank, and distal shank showed that midthigh sensor metrics were associated with optical motion capture thrust measurements while having less variability than midshank sensors [33]. Another study using sensors on the tibial tubercles and dorsal surface of the foot found greater peak varus thrust in the severe OA group when compared with their early-stage OA group [34]. Iwama et al [35] assessed the correlation between peak KAM and peak-to-peak difference of acceleration in the medial-lateral axis using sensors on the sternum, pelvis, thighs, and shanks and found that the shank sensor had the highest correlation (*R*=0.57). Wang et al [36] trained 2 machine learning algorithms using raw IMU data from sensors on the bilateral lateral malleoli to provide an accurate, real-time estimation of KAM during walking. The models—XGBoost and an artificial neural network—were trained to estimate KAM from a data set of both healthy individuals and those with knee OA, with both models having an *R*^2^ value of approximately 0.95 [36]. Finally, single sensors on top of the shoes were used to estimate the FPA with a maximum mean error of approximately 2.6° [37]. These approaches show promise for the use of wearables for accurate estimations of these important gait parameters in people with knee OA with the potential for gait retraining interventions that can directly target these parameters. However, further validation of these approaches in free-living conditions is required before they can be implemented in future interventions.

Gait retraining to alter parameters related to OA progression is a natural follow-up to the aforementioned work. In knee OA, gait retraining typically aims to decrease the KAM [36,65,66], a parameter linked to the severity and progression of knee OA [67,68]. Karatsidis et al [38] used Microsoft HoloLens, an augmented reality headset, to provide feedback on FPA from 7 IMUs (on the pelvis, thighs, shanks, and feet) and found similar effectiveness between this approach and a laboratory approach (ie, projection screen in front of the participant) based on steps falling within a +2° to -2° targeted range. Furthermore, IMU-based FPA estimates closely matched those obtained from optical motion capture (overall root mean square difference of 2.38°) [38]. Xia et al [39] developed a shoe with an IMU-embedded insole and vibration motor to provide haptic feedback directly during walking to correct FPA, with participants successfully adopting 5 different FPA walking patterns after training. Although all these prior studies attempted to indirectly reduce KAM by altering other parameters (eg, FPA), some of the approaches discussed earlier that attempted to directly estimate KAM could potentially be used for gait retraining interventions in the future by adding feedback about this parameter [35,36].

### Assessment of OA Intervention Outcomes

There has also been considerable interest in using wearable technology to remotely monitor data following interventions for OA (Table 4; Table S4 in Multimedia Appendix 1). Lebleu et al [40] used inertial sensors to track improvements in lower limb joint angles before and after administering a genicular nerve blockade in patients with knee OA and found a 9.3° increase in sagittal plane ROM during gait and a 3.3° decrease in pelvic transverse ROM when walking upstairs. In a novel application, Goślińska et al [41] used IMUs to measure proprioception during physical therapy in patients with knee OA to assist in patient evaluation. Wearable sensors are used more often to monitor outcomes in patients undergoing joint replacement surgery. Hsieh et al [42] used a 6-sensor system during the timed up and go test to identify subphases with this task using machine learning for patients with TKA; using preoperative and postoperative data, they achieved a classification accuracy of 92% for segmentation of subphases during the timed up and go test. Inertial sensors have also been used to identify remaining gait asymmetry following a 4-week rehabilitation program in individuals post THA [69]. These studies demonstrate the potential of wearable technologies to monitor functional recovery after joint replacement surgeries in patients with knee or hip OA, potentially identifying individuals who may require additional rehabilitation or other medical care. When combined with patient factors (BMI, anesthesia status, and hemostatic use), data from wearables were used to identify associations between these factors and knee ROM post TKA [43]. Thus, inertial sensors could be used not only to understand how interventions affect biomechanics or movement quality but also how patient factors are related to these outcomes.

**Table 4:** Inertial sensors and assessment of osteoarthritis intervention outcomes.

Study	Population	Sensor	Findings
Bloomfield et al, 2021 [51]	TKA^a^ (n=82)	4 IMU^b^ (MetaMotionR, MBientLab) on thighs and shanks	Using only sensor data and no method of feature selection, random forest model was able to separate responders from maintainers with 93% accuracy
Bloomfield et al, 2019 [50]	TKA (n=68)	4 IMU^b^ on thighs and shanks	Successfully grouped patients using preoperative functional data into high function and low function short-term recovery groups
Bolink et al, 2016 [44]	Healthy (n=30) and THA^c^ (n=36)	Single IMU^d^ (Inertia-Link, MicroStrain) on posterior superior iliac spine	Preoperative differences in gait parameters between low and high function groups disappeared by 3-month postoperative time point
Chiang et al, 2017 [43]	TKA (n=18)	2 IMU^b^ (OPAL, APDM) on thigh and shank	Different range of motion patterns present in patients that received different hemostatic agents shortly after surgery
Di Benedetto et al, 2019 [46]	TKA (n=26)	4 IMU^b^ (Bioval, Movea)	One TKA implant performed better in rotational flexion and freedom than other
Goślińska et al, 2020 [41]	Healthy (n=27) and TKA (n=54)	2 IMU^b^ (Orthyo, Aisens) distal to both greater trochanter and tibial tuberosity	No significantly impact of different rehabilitation programs on affected knee position sense in OA^e^ groups
Grip et al, 2019 [47]	Healthy (n=8), THA (n=15)	5 IMU^d^ (MoLab, AnyMo AB) on pelvis, thighs, and shanks	Large femoral head THA surgery group had greater hip flexion range of motion than traditional THA surgery group
Hsieh et al, 2020 [42]	THA (n=26)	6 IMU^d^ (OPAL) on chest, back, thighs, and shanks	Accuracy >90% in timed up and go subtask segmentation with AdaBoost machine learning technique
Kluge et al, 2018 [49]	Healthy (n=24), TKA (n=24)	2 IMU^d^ (Shimmer3, Shimmer Sensing) on each foot	Wearable-derived metrics consistent with previous literature on gait function in post-TKA populations
Kobsar et al, 2017 [52]	Knee OA (n=39)	4 IMU^d^ (iNEMO inertial module, STMicroelectronics) on foot, shank, thigh, and back	Sensor data were more accurate than patient-reported outcome measures in predicting response to hip strengthening program
Kobsar, and Ferber, 2018 [53]	Knee OA (n=8)	4 IMU^d^ (iNEMO inertial module) on foot, shank, thigh, and back	Average of 84 principal components needed to describe 95% of variance in gait patterns related to improvements in clinical outcomes
Lebleu et al, 2020 [40]	Healthy (n=12), knee OA (n=14)	7 IMU^b^ (x-IMU, x-io Technologies) on waist, thighs shanks, and feet	Cadence and stride time changed significantly after nerve blockade injections, tending toward values of healthy individuals
Menz et al, 2016 [48]	First metatarsophalangeal OA (n=97)	4 IMU^d^ (LEGSys, Biosensics) on thighs and shanks	Orthoses did not produce significant changes on spatiotemporal and kinematic parameters, rocker-sole reduced cadence to small effect and increased % stance time and reduced sagittal plane hip ROM to medium effect
Shah et al, 2019 [45]	THA (n=10) and TKA (n=7)	Single IMU^b^ (Lumo Lift, Lumo Bodytech) on pelvis	Raw data give better understanding than 24-hour summarized data for correlating with patient-reported outcome measures

^a^TKA: total knee arthroplasty.

^b^IMU: inertial measurement unit (with accelerometer, gyroscope, and magnetometer).

^c^THA: total hip arthroplasty.

^d^IMU unit with accelerometer and gyroscope.

^e^OA: osteoarthritis.

Wearable sensor data may provide information about recovery beyond that captured by the subjective measures of change. Bolink et al [44] identified that objective gait parameters capture a dimension of physical function that is distinct from Western Ontario and McMaster Universities Arthritis Index scores in individuals post THA. Although Western Ontario and McMaster Universities Arthritis Index scores improved in patients with both low and high preoperative function at 3-month post THA, gait parameters only improved in those with low preoperative function [44]. This finding that individuals with lower function have more functional improvement to gain from THA highlights the potential of inertial sensors to capture additional insights that are not clear from subjective data alone [44]. Furthermore, Shah et al [45] determined that increasing the sampling frequency of the sensor improves the accuracy of machine learning algorithms in predicting patient-reported outcomes.

Wearable sensors have also been used to compare the outcomes of various OA treatments. Di Benedetto et al [46] used a 4-IMU system (Bioval) to compare kinematic outcomes in patients who underwent TKA using different implants, finding a significant increase in knee flexion in one group. In addition, using sensors on the pelvis, thighs, and shanks, Grip et al [47] found larger ROM during squats, gait, and stair ascent and descent in individuals receiving a THA implant with a larger femoral head than in those who received a conventional implant. IMUs have similarly been used to compare the effects of prefabricated foot orthoses and rocker-sole footwear on spatiotemporal parameters, hip and knee kinematics, and plantar pressure in individuals with OA of the first metatarsophalangeal joint [48]. Using IMUs on the shanks, thighs, and lower back, along with plantar pressure insoles, Menz et al [48] demonstrated that both interventions reduced the peak pressure beneath the first metatarsophalangeal joint and heel, but the rocker-sole footwear additionally reduced the pressure across the second through fifth metatarsophalangeal joints, whereas the orthoses increased the peak pressure under the lesser toes and midfoot. Although this study had a small sample relative to the number of comparisons, it highlights a novel application of wearable technology to study how interventions affect muscle force [70]. In general, the studies discussed above highlight the potential of inertial sensors to provide objective outcomes in clinical trials with relative ease.

With a heterogeneous OA population that may respond differently to interventions, an exciting area of development is in predicting the response to treatment. For example, high preoperative gait function assessed using 2 feet-worn IMUs was predictive of functional decreases post TKA, suggesting that those with lower preoperative function have more to gain [49]. In addition, positive and negative responders can be predicted with an accuracy of up to 89% [49]. Bloomfield et al [50] used IMU data from sensors above and below the knee on participants during the timed up and go test preoperatively to group patients by functional improvement likelihood and to predict expected functional recovery after TKA [51]. Similarly, Kobsar et al [52] classified nonresponders, low responders, and high responders to a 6-week hip and core strengthening program for knee OA with 81.7% accuracy using preintervention data from IMUs on the lower back, thigh, shank, and foot, and similar results were obtained using a simplified 2-sensor system (thigh and back IMU data only). Furthermore, using a subsample of participants, Kobsar et al [53] identified gait pattern changes that were associated with self-reported pain and function outcomes using a novel, subject-specific, machine learning approach, suggesting that machine learning analyses can be used with wearable sensor data in clinically meaningful ways.

Most studies discussed in this section had small sample sizes with some being preliminary in nature. However, these studies demonstrated a wide range of possibilities with the use of wearable sensors to monitor intervention outcomes and predict responses to interventions.

### Sensor Placement and Data Analysis

Given the variety of different parameters and sensor configurations used in studies using inertial sensors in populations with hip and knee OA or joint replacement, there has also been interested in investigating the effect of sensor configuration and data analysis on outcomes (Table 5; Table S5 in Multimedia Appendix 1). For example, Sharifi et al [54] used machine learning to analyze 15 combinations of data from a maximum 7-IMU system (feet, pelvis, shank, and thigh sensors) on individuals with OA and TKA to determine the optimal sensor combination to capture spatiotemporal gait parameters, with the feet-thigh combination having the best overall rank based on normalized absolute percentage error compared with the other sensor combinations. A few of the studies mentioned in this review also incorporated a comparison of different sensor locations into their work [16,33,35,52], with the goal of optimizing the balance between convenience and patient burden (ie, low number of sensors) and valid data.

**Table 5:** Inertial sensors sensor placement and data analysis.

Study	Population	Sensor	Findings
Boekesteijn et al, 2021 [56]	Healthy (n=27), knee OA^a^ (n=25), and hip OA (n=26)	4 IMU^b^ (OPAL, APDM) on feet, lumbar spine, and sternum	Stride length and cadence had strongest effect sizes for both OA groups during turning and dual-task performance during walking
Sharifi et al, 2020 [54]	Knee OA (n=14) and TKA^c^ (n=15)	7 IMU^d^ (Xsens Technologies BV) on pelvis, thighs, shanks, and feet	Feet-thigh sensor combination identified as best for measuring spatiotemporal gait parameters
Teufl et al, 2019 [55]	Healthy (n=24) and THA^e^ (n=20)	7 IMU^b^ (Xsens Technologies BV) on pelvis, thighs, shanks, and feet	Joint angles yielded 97% accuracy in differentiating gate between groups, spatiotemporal metrics gave 87.2% accuracy

^a^OA: osteoarthritis.

^b^IMU: inertial measurement unit (with accelerometer, gyroscope, and magnetometer).

^c^TKA: total knee arthroplasty.

^d^IMU with accelerometer and gyroscope.

^e^THA: total hip arthroplasty.

In addition to various sensor placement combinations, various methods for analyzing inertial sensor data have been explored. Teufl et al [55] trained 2 different support vector machines—one using spatiotemporal gait parameters and one using joint angles, both from a 7-IMU system—to differentiate between impaired and nonimpaired gait using healthy controls and individuals post TKA. Both machines were successful (87.2% and 97.0% accuracy), and hip ROM symmetry was the most important single predictive feature, being roughly 3 times more important than the next feature, pelvic sagittal ROM [55]. In a study of individuals with knee OA, hip OA, and healthy controls, Boekesteijn et al [56] created 4 independent gait domains as a way to reduce the dimensionality of their data set and found the domains containing stride length, cadence, and lumbar sagittal ROM to be the most sensitive to detecting the presence of knee or hip OA. Other studies previously mentioned in this review (Tables 1-4) examined a variety of extracted metrics, with a few using machine learning for feature extraction or outcome prediction [31,32,36,51,53]. These studies provide initial information about how sensor placement and data analysis affect outcomes; however, given the variety of factors used in the current literature, more work is needed in this area to identify the ideal sensor placements and extracted datatypes for specific applications of inertial sensors in lower limb OA.

## Discussion

### Principal Findings

This review sought to examine the use of inertial sensors to assess the movement in the context of hip and knee OA clinical care in patients’ natural environments. We identified various applications of inertial sensors in hip and knee OA that have been published over the past 5 years, including assessment of OA presence and severity, assessment of and intervention on risk factors for OA progression, tracking intervention outcomes, and identifying individuals most likely to respond to interventions. Although further work is needed to validate the findings in real-world environments and determine optimal sensor placement and data analysis methods, the use of inertial sensors for these applications in hip and knee OA could improve opportunities for remote research and clinical care, particularly given the shifting health care landscape resulting from the COVID-19 pandemic [71].

### Comparison With Prior Work

There have been 2 previous reviews of wearable sensors in OA or postarthroplasty populations; however, these focused on very specific applications (gait analysis or postsurgical outcomes), whereas this review sought to assess all current and potential uses of inertial sensors in these populations. A scoping review by Kobsar et al [72] on inertial sensors for gait analysis in individuals with OA identified multiple studies using inertial sensors for this application, with a range of sensor placements and outcomes used among the included studies. Although we similarly identified a range of sensor placements and outcome measures used in the studies included in this review, our results are based on the assessment by Kobsar et al [72] regarding sensor protocols and outcome measures by examining the range of challenges and problems to which wearable sensors can and have been applied, including those beyond gait analysis. Importantly, both reviews identified the need to validate inertial sensor assessment of gait in free-living environments. Another review focused solely on wearable sensors in assessing functional outcome measures after lower extremity arthroplasty and found wearable sensors to be more sensitive than traditional functional outcome measures [73]. Both this review and the current one suggest that more work is needed to understand the clinical relevance of sensor measures.

Finally, we would like to recognize the timeliness of this review within the wider scope of the current research and global events. At the time of writing, the global COVID-19 pandemic is still ongoing [74]. This event accelerated both the adoption of remote health care [75] and the use of digital health technologies for the remote assessment of participants in clinical trials [76]. By rerunning our literature search in March 2021, we were able to capture and include many studies using inertial sensors that were published during the first year of the pandemic. Of the 43 studies included in this review, 24 were published in either 2020 or the first 3 months of 2021. As the landscape of both data collection in general and the management of clinical trials moves outside of the laboratory with inertial sensors and wearable technology, we believe this review adds an important summary of new and current sensor applications to the existing body of literature.

### Limitations

A number of limitations should be considered when interpreting the results of this review. First, although, in this study, we aimed to provide a narrative overview of the various applications of wearable inertial sensors for assessing movement quality in OA populations, the narrative format and change in search scope could have led to a selection bias in the studies included. To mitigate the risk of selection bias, 2 researchers (MJR and KEC) reviewed all identified abstracts from the final search strategy for potential inclusion and additionally reviewed studies selected for inclusion in the earlier searches to determine if they met the updated scope. Second, given the narrative format of this review, the quality of included studies was not assessed. Third, limiting the search to studies published within the past 5 years may have resulted in the exclusion of relevant studies published outside this range. This pragmatic choice was made owing to a significant increase in the number of publications on wearable sensors in recent years to present current results from this rapidly moving field. Fourth, the significant variability in sensor placement across the included studies limited our ability to draw conclusions regarding best practices for specific applications. Finally, this review does not address patients’ and clinicians’ perspectives on wearable technology. The reader is advised to consider stakeholder perspectives when implementing inertial sensors to assess movements in OA populations.

### Future Directions

The results of this review highlight the potential of wearable sensors for remote monitoring of patients with OA and identification of those at risk for whom interventions may be needed. However, this work has primarily been done in relation to walking gait, with relatively few studies examining other types of movement (lunges, stair ascent and descent, squatting, sit-to-stand, and single-leg stance) [29,31,47] commonly experienced during everyday life. In addition, as described by Kobsar et al [72] in a scoping review of inertial sensors for gait analysis in individuals with OA, more work is required in free-living environments. Given the low number of nongait studies and the high prevalence of laboratory-based data collection in the studies included in this review, further work is needed to validate whether inertial sensor data captured from various real-world activities are sensitive to disease initiation and risk of progression and thus could be used for remote monitoring and risk screening.

In addition, we found only a handful of studies focused on training of movement patterns for individuals with OA, and of those we did identify, all focused on the feasibility and validation of gait retraining interventions. Questions remain around the large-scale deployment of inertial sensor—driven gait retraining or similar programs. The conclusions on the efficacy and acceptability of the interventions are of interest. Finally, although a few of the studies included in this review reported good reliability and validity of metrics extracted from inertial sensor data, a wide range of inertial sensor systems and extracted parameters were used in the various applications reviewed here. Continued research into optimal sensor placement to best capture relevant outcomes with minimum burden on the individual patient or participant may encourage the widespread use of these systems to capture biomechanical data in real-world settings.

### Conclusions

Multiple opportunities exist to use inertial sensors to enhance remote health care for hip and knee OA. Within the last 5 years, research using inertial sensors in these populations has focused on the validity and repeatability of measurements, assessment of OA presence and severity, assessment of movement patterns associated with OA progression and gait retraining, assessment of OA intervention outcomes, and sensor placement and data analysis. Although these applications show great promise, further work is needed to investigate the use of inertial sensors in real-world settings, in a variety of activities of daily living, and in larger samples of individuals with hip and knee OA.

## Appendix

Multimedia Appendix 1 Detailed tables on studies included in Results section of paper. Additional information given on study population (demographics data), sensor use and specifications, and application specifics.

## Abbreviations

FPA: foot progression angle
IMU: inertial measurement unit
KAM: knee adduction moment
OA: osteoarthritis
ROM: range of motion
THA: total hip arthroplasty
TKA: total knee arthroplasty
