# Supplementary material for: Inertial Measurement Units and Application for Remote Health Care in Hip and Knee Osteoarthritis: Narrative Review
Source: JMIR Rehabil Assist Technol. 2022 Jun 2;9(2):e33521. doi: 10.2196/33521 (PMC9204569; doi:10.2196/33521)
Supplement: Multimedia Appendix 1 [file rehab_v9i2e33521_app1.docx]

Table S1. Inertial sensors validity and reliability measuring movement.

| **Bravi et al, 2020 [24]** | |
| --- | --- |
| Purpose | - Validity and reliability of single trunk IMU for spatial-temporal gait in recent THA/TKA patients using crutches |
| Study Population | - Healthy individuals: n = 10, age = 43.40 ± 9.45, sex(M:F) = 6:4, BMI = 24.73 ± 3.58 - THA/TKA patients: n = 20, THA:TKA = 10:10, age = 69.05 ± 11.18, sex(M:F) = 14:6, BMI = 28.81 ± 3.05 |
| Sensor Details | - Single IMU^a^ (G-WALK, BTS Bioengineering, Milan, Italy) placed on lower trunk - Sampling rate = 100 Hz, accelerometer ± 8g, gyroscope ± 250 deg/s, magnetometer ± 1200 uT |
| Application Specifics | - 5 gait trials at self-selected pace - Trigger box used for synchronization with operator verifying comparable and verified steps/gait events - Tested mean speed, cadence, stride length, stride duration, and stance, swing, and double support phases as a % of gait cycle time using intraclass correlation coefficient (2,k) |
| Findings | - Single IMU was found to be reliable and valid in altered gait patterns using crutches - Major limitation in IMU gait cycle phase recognition |
| **Charlton et al, 2019 [18]** | |
| Purpose | - Validity and reliability of single shoe-embedded sensor to measure FPA |
| Study Population | - Healthy individuals: n = 20, age = 28.3 ± 10.1, sex(M:F) = 12:8, BMI = 23.1 ± 2.9 |
| Sensor Details | - IMU^a^ (MPU-9150, InvenSense, CA, USA) embedded into shoe sole underneath heel - Sampling rate = 100 Hz, accelerometer ± 4 g, gyroscope ± 500 deg/s, magnetometer ± 1200 uT |
| Application Specifics | - Over-ground walking both in lab and on outdoor track - Validated sensor against motion capture in the lab scenario |
| Findings | - Sensor were valid and provided good to excellent reliability in measurement of FPA in over-ground walking |
| **Fennema et al, 2019 [17]** | |
| Purpose | - Evaluate repeatability of IMU joint angles using robot controller and leg phantom |
| Study Population | - Anthropomorphic leg phantom with 6 degree of freedom robotic controller |
| Sensor Details | - 2 IMU^a^ (﻿MetaMotionR, mbientlab, San Francisco, CA, USA) placed on thigh and shank - Sample rate = 25 Hz |
| Application Specifics | - Two sensor placement setups were tested, one with sensors on midline of thigh and shank, one with sensors on the lateral side of thigh and shank - Sensor data validated against optical motion capture |
| Findings | - Both IMU setups gave acceptable repeatability in ROM measurements |
| **Hafer et al, 2020 [21]** | |
| Purpose | - Validate IMUs for measuring spatiotemporal gait parameters and knee ROM across different age and disability groups |
| Study Population | - Healthy individuals (young): n = 10, age = 27.9 ± 4.7, sex(M:F) = 5:5 - Healthy individuals (older): n = 10, age = 72.3 ± 3.3, sex(M:F) = 5:5 - Knee OA: n = 9, age = 69.2 ± 4.5, sex(M:F) = 5:4 |
| Sensor Details | - 4 IMU^a^ (OPAL, APDM, Portland, OR, USA) on dorsal foot, lateral shank and thigh, and lower back - Sampling rate = 128 Hz, accelerometer ±16 g, gyroscope ±2000 deg/s, magnetometer ±8 Gauss |
| Application Specifics | - Participants completed 10 walking trials at 3 different speeds - Spatiotemporal parameters and knee ROM were analyzed |
| Findings | - Minimal IMU setup and reproducible methods were shown to accurately capture fundamental gait metrics |
| **Ismailidis et al, 2020c [22]** | |
| Purpose | - Validate inertial system for measuring kinematic differences between hip OA and healthy individuals |
| Study Population | - Healthy individuals: n = 45, age = 66.6 ± 7.4, sex(M:F) = 16:29, BMI = 25.0 ± 4.1 - Hip OA: n = 22, age = 66.3 ± 10.2, sex(M:F) = 12:10, BMI = 27.3 ± 3.3 |
| Sensor Details | - 7 IMU^a^ (RehaGait, Hasomed, Magdeburg, Germany) on pelvis and bilaterally on feet, shanks, and thighs |
| Application Specifics | - Participants completed one walking trial over 20m at a self-selected speed - Spatiotemporal kinematic parameters calculated for hip, knee, and ankle |
| Findings | - Validated commercial IMU system against previous literature on marker-based data differences between hip OA and healthy individual groups |
| **Ismailidis et al, 2021 [23]** | |
| Purpose | - Investigate difference in IMU measures between legs and groups with commercial system |
| Study Population | - Healthy individuals: n = 46, age = 66.8 ± 7.4, sex(M:F) = 16:30, BMI = 25 ± 4.0 - Knee OA: n = 22, age = 65.9 ± 9.1, sex(M:F) = 12:10, BMI = 27.8 ± 3.6 |
| Sensor Details | - 7 IMU^a^ (RehaGait, Hasomed, Magdeburg, Germany) on pelvis and bilaterally on feet, shanks, and thighs |
| Application Specifics | - Participants completed one walking trial over 20m at a self-selected speed - Data obtained from the first two and last two steps were excluded from the analysis - Kinematic gait parameters between affected and unaffected side in patients with unilateral knee OA and between patients with OA and healthy controls |
| Findings | - Sensors were able to detect differences between affected and unaffected side in individuals with knee OA, and between individuals with knee OA and healthy individuals |
| **Konrath et al, 2019 [20]** | |
| Purpose | - Estimate KAM and tibiofemoral joint contact force during daily living |
| Study Population | - Healthy individuals: n = 8, age = 59 ± 8, sex(M:F) = 6:2, |
| Sensor Details | - 17 IMU^a^ (﻿Xsens Awinda, Xsens Technologies BV, Enschede, Netherlands) on head, sternum, pelvis, and bilaterally on thighs, shanks, feet, shoulders, upper arms, forearms, and hands - Sampling rate = 60 Hz |
| Application Specifics | - Gait analyses and stair climbing performed with sensor system and marker-based motion capture |
| Findings | - Moderate to strong Pearson correlation coefficients found in KAM and tibiofemoral joint contact force calculations |
| **Zügner et al, 2019 [26]** | |
| Purpose | - Validate IMUs for measuring pelvic tilt and hip and knee flexion in THA patients |
| Study Population | - THA patients: n = 49, age = 71 (51-80), sex(M:F) = 25:24, BMI = 28.7 (20-44) |
| Sensor Details | - 6 IMU^b^ (GaitSmart, Dynamic Metrics Ltd., Hertfordshire, UK) placed bilaterally under iliac crests, thighs, and shanks - Sampling rate = 102.4 Hz |
| Application Specifics | - Participants completed one walking test at a self-selected speed over 6m |
| Findings | - No difference in mean pelvic tilt or knee flexion angles measured by the IMU versus optical motion capture system - IMU system recorded lower hip flexion angles (34.0-34.4° vs. 36.7-37.7°, respectively; p < 0.001) |
| IMU^a^ = inertial measurement unit with accelerometer, gyroscope, and magnetometer; IMU^b^ = inertial measurement unit with accelerometer and gyroscope; OA = osteoarthritis; BMI = body mass index; TJA = total joint arthroplasty; THA = total hip arthroplasty; TKA = total knee arthroplasty; ROM = range of motion; | |

Table S2. Inertial sensors and assessment of osteoarthritis presence and severity.

| **Barrois et al, 2016 [27]** | |
| --- | --- |
| Purpose | - Discriminate levels of OA severity using IMU data |
| Study Population | - Healthy individuals: n = 12, age = 63.2 ± 17.1, BMI = 25.2 ± 4.6 - Knee/hip OA moderate impairment group: n = 24, age = 70.5 ± 9.5, BMI = 26.8 ± 5.7, WOMAC = 14.1 ± 10 - Knee/hip OA severe impairment group: n = 24, age = 70.5 ± 14.9, BMI = 28.2 ± 5.7, WOMAC = 62.58 ± 14.0 |
| Sensor Details | - 4 IMU^a^ (MTw, Xsens Technologies B.V., Culver City, CA, USA) on both feet, lower back, and head - Sampling rate = 100 Hz, accelerometer ± 16 g, gyroscope ±1200 deg/s |
| Application Specifics | - 2 laps of 10-meter walking course at self-selected pace - Analyzed mean and root mean square signals from all sensors (60 parameters total) without step detection |
| Findings | - Discrimination capacity was found in 4 of 61 parameters tested – mean and root mean square of norm of horizontal plane acceleration in both feet |
| **De Brabandere et al, 2020 [36]** | |
| Purpose | - Estimate hip and knee joint loading using sensors in a mobile phone with machine learning |
| Study Population | - End-stage Hip OA: n = 20, cohort characteristics not given |
| Sensor Details | - Single IMU^b^ (Samsung Galaxy J5 2017, Samsung, Seoul, South Korea) inside cell phone, attached to participant’s hip - Sampling rate = 50 Hz |
| Application Specifics | - Participants performed 9 different exercises including walking, stairs, sitting, lunges, single leg standing, and single leg squats - Machine learning to predict knee and hip contact forces estimated using musculoskeletal model from IMU data |
| Findings | - Overall error was too large to be applicable in a clinical context, but the authors were able to train and evaluate a machine learning pipeline using only the mobile phone sensor as input and using data from patient individuals |
| **Dindorf et al, 2020 [37]** | |
| Purpose | - Examine how different sensor data (gait cycle waveforms vs simple descriptive stats vs automatically extracted features) influence machine learning model’s accuracy, interpretability, and clinical relevancy |
| Study Population | - Healthy individuals: n = 27, age = 24.63 ± 2.80, sex(M:F) = 13:14, weight = 70.44 ± 12.56 kg, height = 1.76 ± 0.09 m - THA patients: n = 20, age = 57.79 ± 7.41, sex(M:F) = 7:13, weight = 83.89 ± 17.22 kg, height = 1.73 ± 0.08 m |
| Sensor Details | - 7 IMU^b^ (Awinda, Xsens Technologies BV, Enschede, The Netherlands) on both feet, shanks, thighs, and lower back - Sampling rate = 60 Hz |
| Application Specifics | - IMU data was used to calculate hip, knee, and ankle joint angle waveforms, as well as the global pelvic motion in the sagittal, frontal, and transverse plane - Machine learning to classify healthy participants and those with THA using IMU inputs from 6-minute walk test |
| Findings | - Input representation type significantly determines machine learning models’ interpretability and clinical relevancy - Automatically extracted features gave best accuracy in classifying THA patients and healthy individuals |
| **Ismailidis et al, 2020a [30]** | |
| Purpose | - Investigate the effect walking speed on spatiotemporal parameters and kinematic trajectories |
| Study Population | - Healthy individuals: n = 48, age = 66.6 ± 7.2, sex(M:F) = 18:30, BMI = 25.1 ± 4.0 - Hip OA: n = 24, age = 66.1 ± 10.3, sex(M:F) = 14:10, BMI = 27.5 ± 3.2 |
| Sensor Details | - 7 IMU^a^ (RehaGait, Hasomed, Magdeburg, Germany) on pelvis and bilaterally on feet, shanks, and thighs |
| Application Specifics | - All participants completed one walking trial over 20m at a self-selected speed, and control subjects completed an additional walking trial at a slow self-selected speed - Compared gait parameters between patients with hip OA and healthy controls for normal and matched speeds |
| Findings | - Significant changes in hip and knee kinematics exist between hip OA and healthy individuals in speed matched conditions |
| **Ismailidis et al, 2020b [31]** | |
| Purpose | - Investigate effect of walking speed on kinematic parameters in knee OA |
| Study Population | - Healthy individuals: n = 28, age 68.8 ± 6.5, sex(M:F) = 10:18, BMI = 28.1 ± 3.8 - Knee OA: n = 23, age 66.1 ± 8.9, sex(M:F) = 12:11, BMI = 24.9 ± 3.8 |
| Sensor Details | - 7 IMU^a^ (RehaGait, Hasomed, Magdeburg, Germany) on pelvis and bilaterally on feet, shanks, and thighs |
| Application Specifics | - Gait analyses, spatiotemporal parameters and sagittal plane joint kinematics; compared between healthy and OA groups at different walking speeds |
| Findings | - Significant differences in all spatiotemporal parameters between groups when walking at self-selected speed |
| **Na et al, 2021 [29]** | |
| Purpose | - Investigate relationship between wearable sensor data and self-reported instability in knee OA patients |
| Study Population | - Healthy individuals: n = 13, age = 66.2 ± 6.2, sex(M:F) = 5:8, BMI = 27.6 ± 3.7 - Knee OA: n = 26, age = 65.9 ± 6.1, sex(M:F) = 10:16, BMI = 30.5 ± 5.6 |
| Sensor Details | - 5 IMU^b^ (Noraxon, 3D Myomotion, Scottsdale, AZ, USA) on pelvis, and bilateral thighs and shanks |
| Application Specifics | - Participants walked at controlled gait speeds of 1.0 m/s as well as self-selected gait speeds over 10m - Knee instability measured via questionnaire |
| Findings | - Linear acceleration (significant) and jerk (insignificant) were negatively associated with self-reported instability |
| **Odonkor et al, 2020 [32]** | |
| Purpose | - Determine best sensor-derived gait parameters to discriminate knee OA and lumbar spinal stenosis patients and healthy controls |
| Study Population | - Healthy individuals: n = 10, age = 61.2 ± 9.9, sex(M:F) = 5:5, BMI = 27.2 ± 4.1 - Knee OA: n = 10, age = 63.9 ± 8.1, sex(M:F) = 4:6, BMI = 33.2 ± 8.4 - Lumbar spinal stenosis: n = 10, age = 70.3 ± 9.4, sex(M:F) = 3:7, BMI = 29.6 ± 4.1 |
| Sensor Details | - 2 IMU^a^ (Shimmer3, Shimmer Sensing, Dublin, Ireland) bilaterally on dorsal surface of foot - Sampling rate = 102.4 Hz |
| Application Specifics | - Three clinically validated walking tests (40-m fast paced walk test, 6-minute walk test, and self-paced walk test) - Spatial and temporal gait metrics analyzed |
| Findings | - Stance and double support ratio were the two most consistent discriminating features between OA and controls, and metrics from the 6-minute walk test had the best discriminative power between OA and controls |
| **Tadano et al, 2016 [33]** | |
| Purpose | - Validate wearable sensor system for measuring OA gait parameters |
| Study Population | - Healthy individuals: n = 8, age = 22.9 ± 0.8, BMI = 23.0 ± 1.6 - Knee OA: n = 10, age = 68.7 ± 4.1, BMI = 23.5 ± 2.5 |
| Sensor Details | - 7 IMU^b^ (H-Gait system, Laboratory of Biomechanical Design Hokkaido University, Sapporo, Japan) on pelvis and bilateral thighs, shanks, and feet - Sampling rate = 100 Hz |
| Application Specifics | - Participants walked 7m at a comfortable speed - Spatiotemporal parameters and kinematics of the hip, knee, and ankle joints |
| Findings | - Angle between knee trajectories was nearly twice as large in OA individuals compared to healthy controls |
| **Tanimoto et al, 2017 [28]** | |
| Purpose | - Investigate swing limb kinematics in knee OA |
| Study Population | - Healthy individuals: n = 11, age = 66.0 [62.5-73.5], sex(M:F) = 2:9, BMI = 21.6 ± 2.5 - Knee OA: n = 12, age = 73.0 [71.5-73.0], sex(M:F) = 2:10, BMI = 23.4 ± 2.5 |
| Sensor Details | - 1 IMU^b^ (MVP-RF8-GC-500, Microstone, Saku, Japan) on anterior shank - Sampling rate = 100 Hz, accelerometer ± 20 m/s^2^, gyroscope ± 500 deg/s |
| Application Specifics | - Participants walked on instrumented treadmill at self-selected speed for 10 minutes - Kinematic parameters analyzed related to peak shank angular velocity during the swing phase |
| Findings | - Found no differences between two groups for any parameters for peak shank angular velocity |
| **Van der Straaten et al, 2020a [34]** | |
| Purpose | - Differentiate healthy controls and knee OA in functional movement tasks |
| Study Population | - Healthy individuals: n = 12, age = 59.8 ± 7.0, sex(M:F) = 6:6, BMI = 25.1 ± 3.4 - Knee OA: n = 19, age = 65.1 ± 5.2, sex(M:F) = 12:7, BMI = 26.0 ± 2.2 |
| Sensor Details | - 15 IMU^a^ (MVN BIOMECH Awinda, Xsens Technologies BV, Enschede, Netherlands) on the pelvis, sternum, and forehead, as well as bilateral thighs, shanks, feet, forearms, upper arms, and superior borders of the scapulae - Sampling rate = 60 Hz |
| Application Specifics | - Participants completed 5 repetitions of 6 tests including walking, forward lunge, sideward lunge, stair ascent and descent, single leg squat, and sit-to-stand - Compared joint kinematics and angles |
| Findings | - Individuals with knee OA walked with significantly less trunk rotation, less internal pelvic rotation during stance to swing, and reduced knee flexion among other discriminating differences |
| **Van der Straaten et al, 2020b [35]** | |
| Purpose | - Differences in joint kinematics and center of mass displacement in healthy and knee OA |
| Study Population | - Healthy individuals: n = 12, age = 59.8 ± 7.0, sex(M:F) = 6:6, BMI = 25.1 ± 3.4 - Knee OA: n = 19, age = 65.1 ± 5.2, sex(M:F) = 12:7, BMI = 26.0 ± 2.2 |
| Sensor Details | - 15 IMU^a^ (MVN BIOMECH Awinda, Xsens Technologies BV, Enschede, Netherlands) on the pelvis, sternum, and forehead, as well as bilateral thighs, shanks, feet, forearms, upper arms, and superior borders of the scapulae - Sampling rate = 60 Hz |
| Application Specifics | - Participants completed five repetitions of the unipodal stance task - Compared center of mass displacement and joint kinematics directly derived from inertial sensor system |
| Findings | - Knee OA individuals had more lateral trunk lean towards contralateral leg and more hip flexion throughout performance of unipodal stance task |
| OA = osteoarthritis; IMU = inertial measurement unit; BMI = body mass index; WOMAC = Western Ontario and McMaster Universities Arthritis Index; IMU^a^ = inertial measurement unit with accelerometer, gyroscope, and magnetometer; IMU^b^ = inertial measurement unit with accelerometer and gyroscope; THA = total hip arthroplasty; TKA = total knee arthroplasty; ROM = range of motion; KAM = knee adduction moment | |

Table S3. Inertial sensors and assessment of movement patterns associated with osteoarthritis progression and gait retraining.

| **Costello et al, 2020 [41]** | | |
| --- | --- | --- |
| Purpose | - Compare data from single wearable inertial sensor to optical motion capture for measuring varus thrust | |
| Study Population | - Knee OA: n = 26, age = 64.5 ± 8.4, sex(M:F) = 10:16, BMI = 28.6 ± 4.7 | |
| Sensor Details | - 3 IMU^a^ (Trigno IM Sensors, Delsys, Inc., Natick, MA, USA) attached to lateral mid-thigh, lateral mid-shank, and lateral distal shank - Sampling rate = 148 Hz, accelerometer = ± 16 g, gyroscope = ± 2000 deg/s, magnetometer = ± 1000 uT | |
| Application Specifics | - Participants walked in lab at a self-selected “purposeful” pace and at a fast pace - 4-6 clean force plate foot strikes collected for each foot | |
| Findings | - Measures from single leg inertial sensors were associated with surrogate measures of varus thrust, and mid-thigh adduction velocity was significantly associated with peak EKAM | |
| **Ishii et al, 2020 [42]** | | |
| Purpose | - Investigate correlation between lateral thrust and medial meniscus extrusion | |
| Study Population | - Knee OA: n = 44, age = 68.9 ± 9.6, sex(M:F) = 22:22, BMI = 25.1 ± 3.1 | |
| Sensor Details | - 2 IMU^b^ (WAA-010, ATR-Promotions, Kyoto, Japan) placed on the tibial tubercle and the dorsal surface of the foot - Sampling rate = 100 Hz | |
| Application Specifics | - Participants completed two walking trials over 10m at a comfortable speed - Lateral thrust defined as the first lateral acceleration peak in the tibial tubercle IMU | |
| Findings | - Positive correlation between lateral thrust and change in medial meniscus extrusion | |
| **Iwama et al, 2021 [43]** | | |
| Purpose | - Estimate KAM using a single IMU sensor | |
| Study Population | - Knee OA: n = 22, age = 68.5 ± 6.4, sex(M:F) = 3:19, BMI = 22.3 ± 2.6 | |
| Sensor Details | - 6 IMU^b^ (TSND151, ATR-Promotions, Kyoto, Japan) on pelvis, sternum, and bilaterally on shanks and thighs - Sampling rate = 50 Hz (13 cases) or 200 Hz (9 cases), accelerometer ± 4 g | |
| Application Specifics | - Traditional gait analysis performed with motion capture system, force plates to collect ground reaction forces - Analyzed a self-defined “thrust acceleration” as the acceleration peak in IMU local frame immediately after heal contact | |
| Findings | - Moderate correlation found between thrust acceleration and KAM, with values from shank IMU being best correlated | |
| **Karatsidis et al, 2018 [50]** | | |
| Purpose | - Validate wearable visual feedback in retraining foot progression angle | |
| Study Population | - Healthy individuals: n = 11, age = 28.26 ± 4.55, sex(M:F) = 4:7, BMI = 24.5 ± 2.52 | |
| Sensor Details | - 7 IMU^a^ (MTw, Xsens Technologies B.V., Culver City, CA, USA) on both feet, lower back, and head - Sampling rate = 60 Hz, accelerometer ± 16 g, gyroscope ± 2000 deg/s, magnetometer ± 1.9 Gauss | |
| Application Specifics | - Validated feet sensors for measuring FPA against optical motion capture - Visual feedback was provided for user to walk at 5 different foot progression angles, 15, 10 and 5 degrees toe-out, 0 degrees straight toes, and 5 degrees toe-in | |
| Findings | - High accuracy and repeatability of FPA measures - Feedback effectiveness for FPA was similar between wearable and laboratory feedback setups | |
| **Wang et al, 2020 [44]** | | |
| Purpose | - Estimate KAM during walking using wearable sensors and machine learning | |
| Study Population | - Healthy individuals: n = 12, age = 25.4 ± 3.5, sex(M:F) = 8:4, BMI = 22.1 ± 2.8 - Knee OA: n = 78, age = 59.7 ± 7.1, sex(M:F) = 33:45, BMI = 23.0 ± 3.8 | |
| Sensor Details | - 2 IMU^b^ (DA14583, Dialog Semiconductor, Reading, UK) on bilateral malleoli - Sampling rate = 100 Hz, accelerometer ± 4 g, gyroscope ± 2000 deg/s | |
| Application Specifics | - Trained machine learning algorithms using KAM estimations from optical motion capture and force plate data to predict KAM with only raw IMU data inputs | |
| Findings | - Both XGBoost and artificial neural network were highly accurate (R^2^ ~ 0.95) in predicting KAM using IMU input | |
| **Wouda et al, 2021 [45]** | | |
| Purpose | - Estimate FPA with single foot-worn IMU | |
| Study Population | - Healthy individuals: n = 5, age = 25.2 ± 4.2, sex(M:F) = 5:0, BMI = 24.1 ± 3.4 | |
| Sensor Details | - 2 IMU^a^ (MTw Awinda, Xsens Technologies BV, Enschede, Netherlands) on dorsal surface of feet - Sampling rate = 100 Hz | |
| Application Specifics | - Sensor calibration consisted of a static trial and dynamic trial where participants remained still for five seconds with a 0° FPA and walked for four steps with a 0° FPA, respectively - Participants completed three sets of twelve walking trials, including one set with normal FPA, one set with positive (toe-out) FPA, and one set with negative (toe-in) FPA | |
| Findings | - Good correlation coefficients (r^2^ > 0.7) seen for all gait conditions, inertial system found to be able to significantly discriminate between conditions | |
| **Xia et al, 2020 [51]** | | |
| Purpose | - Feasibility study on haptic feedback-sensorized shoe for FPA modification | |
| Study Population | - Healthy individuals: n = 10, age = 27.4 ± 3.2 | |
| Sensor Details | - IMU^a^ (custom made) embedded in sole of shoe | |
| Application Specifics | - Used walking direction from previous steps and angle of shoe to estimate foot progression angle and decide to provide feedback or not - Participants were trained to adopt FPA of 10 degrees toe-in, 0 degrees straight toes, and 10, 20, and 30 degrees toe-out | |
| Findings | - Participants were able to respond to feedback and adopt all target FPA conditions | |
| IMU^a^ = inertial measurement unit with accelerometer, gyroscope, and magnetometer; IMU^b^ = inertial measurement unit with accelerometer and gyroscope; BMI = body mass index; THA = total hip arthroplasty; TKA = total knee arthroplasty; ROM = range of motion; KAM = knee adduction moment | | |
|  |  |  |

Table S4. Inertial sensors and assessment of osteoarthritis intervention outcomes.

| **Bloomfield et al, 2021 [65]** | |
| --- | --- |
| Purpose | - Apply machine learning to multivariate inertial sensor data to predict functional recovery pre-TKA |
| Study Population | - Training set: TKA n = 68, age = 67.5 ± 9.8, sex(M:F) = 34:34, BMI = 33.5 ± 6.0 - Testing set: TKA n = 14, age = 68.4 ± 10.1, sex(M:F) = 9:5, BMI = 33.5 ± 5.8 |
| Sensor Details | - 4 IMU^a^ (MetaMotionR, MBientLab, San Francisco, CA, USA) one on anterior of each thigh and shank - Sampling rate = 25 Hz |
| Application Specifics | - 3 trials of timed up and go test were recorded at preoperative clinical appointment as well as at 2 weeks, 6 weeks, 3 months, 6 months, and 1 year follow-up appointments - Orientation of leg segments were extracted and used to calculate joint-specific and spatiotemporal metrics |
| Findings | - Using only sensor data and no method of feature selection, random forest model was able to separate responders from maintainers with 93% accuracy |
| **Bloomfield et al, 2019 [64]** | |
| Purpose | - Examine functional parameters influencing recovery outcomes in TKA patients |
| Study Population | - TKA: n = 68, age = 67.5 ± 9.8, sex(M:F) = 34:34, BMI = 33.5 ± 6.0 |
| Sensor Details | - 4 IMU^a^ (MetaMotionR, MBientLab, San Francisco, CA, USA) one mounted above and below each knee - Sampling rate = 25 Hz |
| Application Specifics | - 3 trials of timed up and go test were recorded at preoperative clinical appointment as well as at 2-, 6-, and 12-week follow-up appointments - Extracted functional and spatiotemporal metrics |
| Findings | - Successfully grouped patients using preoperative functional data into high function and low function short-term recovery groups |
| **Bolink et al, 2016 [57]** | |
| Purpose | - Compare outcomes of THA between groups with low and high self-reported levels of pre-operative physical function |
| Study Population | - Healthy individuals: n = 30, age = 61.0 ± 5.6, sex(M:F) = 18:12, BMI = 24.8 ± 2.8 - THA: n = 36, age = 63.9 ± 9.8, sex(M:F) = 18:18, BMI = 26.3 ± 3.5 |
| Sensor Details | - IMU^b^ (Inertia-Link, ﻿MicroStrain, Cary, NC, USA) attached to posterior superior iliac spine overlying S1 - Sample rate = 100 Hz, accelerometer ± 5 g, gyroscope ± 300 deg/s |
| Application Specifics | - Gait detection and spatiotemporal metric extraction from sensor data |
| Findings | - Preoperative differences in gait parameters between low and high function groups disappeared by 3-month post-operative time point |
| **Chiang et al, 2017 [56]** | |
| Purpose | - Estimate knee ROM using sensors in post-TKA population |
| Study Population | - TKA: n = 18 |
| Sensor Details | - 2 IMU^a^ (OPAL, APDM, Portland, OR, USA) mounted on anterior side of thigh and shank - Sample rate = 40 Hz, accelerometer ± 6 g, gyroscope x and y axes ± 2000 deg/s, gyroscope z axis ± 1500 deg/s |
| Application Specifics | - Walking and knee bending exercises performed - Correlation analysis conducted between knee ROM and patient health factors |
| Findings | - Found different ROM in patients that received different hemostatic agents shortly after surgery |
| **Di Benedetto et al, 2019 [59]** | |
| Purpose | - Pilot study on kinematic analysis of different TKA groups based on implant used |
| Study Population | - TKA group 1: n = 12, age = 70.25, sex(M:F) = 4:8 - TKA group 2: n = 14, age = 71.75, sex(M:F) = 6:8 |
| Sensor Details | - 4 IMU^a^ (Bioval, Movea, France) |
| Application Specifics | - Sensors used to measure knee rotation in different planes |
| Findings | - One implant performed better in rotational flexion and freedom than the other |
| **Goślińska et al, 2020 [53]** | |
| Purpose | - Assess usefulness of sensors in evaluating knee joint position sense in two different short-term rehabilitation programs |
| Study Population | - Healthy individuals: n = 27, age = 63.0 ± 6.6, BMI = 25.9 ± 3.8 - Knee OA group 1: n = 27, age = 65.0 ± 7.4, BMI = 27.3 ± 3.8 - Knee OA group 2: n = 27, age = 66.1 ± 4.7, BMI = 27.6 ± 4.0 |
| Sensor Details | - 2 IMU^a^ (Orthyo, Aisens, Poznan, Poland) placed 5-15 cm distal to both greater trochanter and tibial tuberosity |
| Application Specifics | - Knee joint proprioception tasks completed by participants while wearing sensors |
| Findings | - Neither rehabilitation program significantly affected knee position sense in OA groups |
| **Grip et al, 2019 [60]** | |
| Purpose | - Pilot study to compare ROM in lower body joints during squats, gait, and stair walking |
| Study Population | - Healthy individuals: n = 8, age = 45 ± 12, sex(M:F) = 8:0, BMI = 23 ± 2 - Traditional THA group: n = 6, age = 56 ± 9, sex(M:F) = 6:0, BMI = 28 ± 2 - Large femoral head THA group: n = 9, age = 49 ± 9, sex(M:F) = 9:0, BMI = 27 ± 4 |
| Sensor Details | - 5 IMU^b^ (MoLab, AnyMo AB, Umea, Sweden) placed on posterior pelvis, right and left thigh, and right and left shank - Sampling rate = 128 Hz, accelerometer ± 10 g, gyroscope ± 300 deg/s |
| Application Specifics | - 5 trials each of squatting, walking 9 meters, and going up and down a flight of stairs - Kinematic outcomes and ROMs for each joint were calculated |
| Findings | - Large femoral head group had significantly greater hip flexion ROM than traditional THA group |
| **Hsieh et al, 2020 [54]** | |
| Purpose | - Develop a subtask segmentation approach for timed up and go test using inertial sensors |
| Study Population | - THA: n = 26, age = 69.15 ± 6.71, sex(M:F) = 5:21 |
| Sensor Details | - 6 IMU^b^ (OPAL, APDM, Portland, OR, USA) on chest, lower back, each thigh, and each shank - Sample rate = 128 Hz |
| Application Specifics | - 3 trials of 5- and 10-meter TUG test at preoperative, postoperative, 2-week, and 6-week postoperative time points - Test was divided into 6 lower limb subtasks |
| Findings | - Found >90% accuracy in subtask segmentation with AdaBoost machine learning technique |
| **Kluge et al, 2018 [63]** | |
| Purpose | - Examine possibility of using sensor system to accurately assess gait function post-TKA |
| Study Population | - Healthy individuals: n = 24, age = 62.3 ± 9.7, sex(M:F) = 8:16, BMI = 24.9 ± 4.1 - TKA: n = 24, age = 64.0 ± 11.0, sex(M:F) = 8:16, BMI = 31.3 ± 6.8 |
| Sensor Details | - 2 IMU^b^ (Shimmer3, Shimmer/Realtime Technologies, Dublin, Ireland) attached laterally to each shoe - Sample rate = 102.4 Hz, accelerometer ± 8 g, gyroscope ± 500 deg/s |
| Application Specifics | - Participants completed 4 laps of 10-meter walking course - Spatiotemporal gait metrics extracted |
| Findings | - System was successful, wearable-derived metrics were consistent with previous literature in post-TKA populations |
| **Kobsar et al, 2017 [66]** | |
| Purpose | - Examine if pre-intervention sensor data and patient reported outcome measures can predict response to hip strengthening program |
| Study Population | - Knee OA: n = 39, age = 59 ± 8, BMI = 26.6 ± 3.8 |
| Sensor Details | - 4 IMU^b^ (iNEMO inertial module, STMicroelectronics, Geneva, Switzerland) custom developed by Alberta Centre for Advanced MNT Products (Calgary, Canada) on foot, shank, thigh, and back on most affected side of knee OA - Sample rate = 100 Hz, accelerometer ± 16 g, gyroscope ± 2000 deg/s |
| Application Specifics | - 60s of gait data collected on instrumented treadmill - Analyzed acceleration data during gait cycles |
| Findings | - Thigh sensor was most effective single sensor in determining response to hip strengthening exercise intervention, but combinations with back and shank provided greatest accuracy - Sensor data was more accurate than patient reported outcome measures own |
| **Kobsar et al, 2018 [67]** | |
| Purpose | - Identify subject-specific changes in gait patterns related to improvements in clinical outcomes |
| Study Population | - 4 IMU^b^ (iNEMO inertial module, STMicroelectronics, Geneva, Switzerland) on foot, shank, thigh, and back on most affected side of knee OA - Sample rate = 100 Hz, accelerometer ± 16 g, gyroscope ± 2000 deg/s |
| Sensor Details | - Knee OA: n = 8, age = 58 ± 5, sex(M:F) = 4:4, BMI = 25.3 ± 4.8 |
| Application Specifics | - 100 gait cycles collected per session on instrumented treadmill - Linear acceleration data analyzed during gait cycles with PCA |
| Findings | - Average number of principal components to describe 95% of total variance was 84 |
| **Lebleu et al, 2020 [52]** | |
| Purpose | - Assessing gait and stair kinematic changes after genicular nerve blockade in knee OA |
| Study Population | - Healthy individuals: n = 12, age = 50.6 ± 11.9, sex(M:F) = 5:7 - Knee OA: n = 14, age = 64.5 ± 11.3, sex(M:F) = 0:14 |
| Sensor Details | - 7 IMU^a^ (x-IMU, x-io Technologies, Bristol, UK) on waist at L5, middle of both thighs and shanks, and dorsal side of feet - Sample rate = 128 Hz, accelerometer ± 6 g, gyroscope ± 2000 deg/s, magnetometer ± 8.1 G |
| Application Specifics | - Different walking and stair tasks - Analyzed spatiotemporal metrics and joint ROM |
| Findings | - Cadence and stride time changed significantly after injections tending towards values of healthy individuals |
| **Menz et al, 2016 [61]** | |
| Purpose | - Study hip and knee kinematics of first metatarsophalangeal OA individuals using foot orthoses and rocker-sole footwear |
| Study Population | - Orthoses group (first metatarsophalangeal OA): n = 51, age = 57.0 ± 11.2, sex(M:F) = 28:23, BMI = 29.2 ± 4.8 - Rocker-sole group (first metatarsophalangeal OA): n = 46, age = 56.5 ± 11.1, sex(M:F) = 18:28, BMI = 28.4 ± 4.5 |
| Sensor Details | - 4 IMU^a^ (LEGSys, Biosensics, Newton, MA, USA) on each thigh and shank |
| Application Specifics | - 4 walking trials of 8-meter distance, only analyzing steady state walking patterns - Spatiotemporal gait metrics and sagittal plane ROM were analyzed |
| Findings | - Orthoses did not produce significant changes on spatiotemporal and kinematic parameters - Rocker-sole reduced cadence to small effect and increased %stance time and reduced sagittal plane hip ROM to medium effect |
| **Shah et al, 2019 [58]** | |
| Purpose | - Study effect of increased frequency of data collection on correlation to patient reported outcome measure data |
| Study Population | - THA/TKA: n = 17, THA:TKA = 10:7, age = 63 ± 6.46, sex(M:F) = 7:10, BMI = 28.51 ± 6.26 |
| Sensor Details | - IMU^a^ (Lumo Lift, Lumo Bodytech, Mountain View, CA, USA) worn on pelvis |
| Application Specifics | - Patients wore device during perioperative period, up to 8 weeks postoperative - Metrics recorded: cadence, bounce, braking, drop/roll, pelvic rotation/yaw, and pelvic tilt/pitch |
| Findings | - Raw data give better understanding than 24-hour summarized data, while predictor signals become stronger as more time passes post-TJA |
| IMU^a^ = inertial measurement unit with accelerometer, gyroscope, and magnetometer; IMU^b^ = inertial measurement unit with accelerometer and gyroscope; THA = total hip arthroplasty; TKA = total knee arthroplasty; TJA = total joint arthroplasty; BMI = body mass index; ROM = range of motion; | |

Table S5. Inertial sensors sensor placement and data analysis.

| **Boekestejin et al, 2021 [70]** | |
| --- | --- |
| Purpose | - Investigate turning and dual-task performance using factor analysis between healthy individuals and knee OA |
| Study Population | - Healthy individuals: n = 27, age = 66 [63-68], sex(M:F) = 13:14, BMI = 25.7 [24.6-26.8] - Knee OA: n = 25, age = 64 [61-67], sex(M:F) = 12:13, BMI = 28.5 [26.9-30.1] - Hip OA: n = 26, age = 64 [61-66], sex(M:F) = 17:9, BMI = 28.1 [26.0-30.1] |
| Sensor Details | - 4 IMU^a^ (OPAL, APDM, Portland, OR, USA) on both feet, lumbar spine, and sternum - Sampling rate = 128 Hz |
| Application Specifics | - 2 minutes of walking back and forth along 6-meter trajectory at comfortable speed with and without a secondary cognitive task - Gait parameters extracted from Mobility Lab software such as range of motion of lumbar and trunk and spatiotemporal gait metrics |
| Findings | - Stride length and cadence had strongest effect sizes for both OA groups, lumbar sagittal range of motion showed strong effect size in hip OA vs healthy controls, and parameters reflecting dual-task cost were not sensitive to either OA group |
| **Sharifi et al, 2020 [68]** | |
| Purpose | - Identify optimal IMU sensor locations and pairings to measure spatiotemporal gait parameters |
| Study Population | - Knee OA: n = 14, age = 67 ± 7, sex(M:F) = 10:4 - TKA patients: n =15, age = 68 ± 4, sex(M:F) = 4:11, |
| Sensor Details | - 7 IMU^b^ (Xsens Technologies BV, Enschede, The Netherlands) on pelvis and bilateral thighs, shanks, and feet - Sampling rate = 40 Hz |
| Application Specifics | - Participants performed fifteen walking trials over 5m at three different speeds - Deep neural network used for comparison of IMU data against motion capture |
| Findings | - Feet-thigh sensor combination gave best results followed by feet-shank - Shank sensor consistently gave highest accuracy for temporal characteristics |
| **Teufl et al, 2019 [69]** | |
| Purpose | - Differentiate impaired and non-impaired gait using IMU data |
| Study Population | - Healthy individuals: n = 24, sex(M:F) = 12:12 - THA patients: n = 20, age = 56.9 ± 8.2, sex(M:F) = 7:13, height = 1.74 ± 0.1 m, weight = 82.9 ± 18.9 kg |
| Sensor Details | - 7 IMU^a^ (MTw Awinda, Xsens Technologies BV, Enschede, Netherlands) on pelvis and bilateral thighs, shanks, and feet - Sampling rate: 60 Hz |
| Application Specifics | - Participants walked a 7m path for a maximum of six minutes - Spatiotemporal gait metrics and joint angles used as separate inputs to 2 different support vector machine models to differentiate gait of THA patients and healthy controls |
| Findings | - Joint angles yielded 97% accuracy in differentiating gate - Spatiotemporal metrics gave 87.2% accuracy |
| IMU^a^ = inertial measurement unit with accelerometer, gyroscope, and magnetometer; IMU^b^ = inertial measurement unit with accelerometer and gyroscope; BMI = body mass index; THA = total hip arthroplasty; TKA = total knee arthroplasty | |
